# A chromosome level reference genome for the pecan weevil, *Curculio caryae*

**DOI:** 10.1038/s41597-026-07030-8

**Published:** 2026-03-18

**Authors:** Lindsey C. Perkin, Zachary P. Cohen, Sheina B. Sim, Scott M. Geib, Anna K. Childers, Timothy P. L. Smith, J. Spencer Johnston, Perot Saelao, Charles P.-C. Suh

**Affiliations:** 1 USDA, Agricultural Research Service, Southern Plains Agricultural Research Center, Insect Control and Cotton Disease Research Unit, 2771 F and B Road, College Station, TX 77845 USA; 2 USDA, Agricultural Research Service, U.S. Pacific Basin Agricultural Research Center, Tropical Crop and Commodity Protection Research Unit, 64 Nowelo Street, Hilo, Hawaii 96720 USA; 3 USDA, Agricultural Research Service, Beltsville Agricultural Research Center, Bee Research Laboratory, 10300 Baltimore Avenue, Beltsville, MD 20705 USA; 4 USDA, Agricultural Research Service, U.S. Meat Animal Research Center, Genetics and Breeding Research Unit, State Spur 18D, Clay Center, NE 68933 USA; 5 Department of Entomology, Texas A&M University, College Station, TX 77845 USA; 6 USDA, Agricultural Research Service, Veterinary Pest Genetics Research Unit, 2700 Fredericksburg Rd., Kerrville, TX 78028 USA

**Keywords:** Genome, Genome informatics

## Abstract

The pecan weevil, *Curculio caryae* (Horn), is an obligate feeder of pecan and native hickory trees (genus *Carya*) throughout North America. Subsequently it is a significant agricultural pest in pecan orchards. In this study, we present a reference quality genome using deep-coverage, ~40x PacBio HiFi genome sequence reads, and chromatin confirmation, Hi-C, scaffolding. The final genome assembly is approximately 2.2 Gb, which was confirmed by flow cytometry. The primary genome scaffolds have an N50 of 132 Mb and a BUSCO completeness of 95.4% [S:94.3%, D:1.1%]. Furthermore, we employed PacBio long-read RNA, Iso-seq, for *de novo* gene annotation, in conjunction with InterProscan to identify approximately 19,000 protein coding genes. Repeat content is extensive, contributing at least >80% of the total genome. This data set provides a valuable resource for comparative genomics and evolutionary studies of an economically impactful group of insect pests that currently lack extensive genomic resources.

## Background & Summary

*Curculionidae* (Coleoptera) is a highly diverse family of insects with over 6,000 genera and more than 97,000 described species (and counting), making it one of the most speciose animal taxa on the planet^1^. Despite the cosmopolitan distribution, incredible diversity and significant impact on ecosystems and agriculture, there remains a relative dearth of genomic information available for Curculionidae species^2–11^. In fact, an NCBI data search (https://www.ncbi.nlm.nih.gov/, accessed September 2025) identifies 51 available genomes with variable quality, representing approximately 0.05% of the total diversity within this ubiquitous 175-million-year-old Curculionidae clade^12^.

The pecan weevil, *Curculio caryae* (Horn), is an impactful pest of pecan (*Carya illinoinensis* Koch) and obligate feeder on hickory trees (*Carya spp*.). Its native range coincides with both native trees and commercial pecan orchards in the United States, stretching from New Mexico to the Carolinas and north into Illinois. The pecan weevil causes significant economic damage, exceeding millions of dollars in losses each year^13–15^. The pecan nut is highly susceptible to the pecan weevil, which can damage an entire orchard, limit trade, and reduce profitability^16^. In fact, the presence of pecan weevils in an orchard can condemn the entire harvest as well as prevent international trade. Despite their impact on the commercial pecan industry, very little genetic information is available to inform growers of basic biological processes for managing the pecan weevil.

In this paper we provide an annotated, chromosome-scale reference genome for the pecan weevil, which represents the first published *Curculio* genome. We find that the genome is 2.169 Gb assembled into 13 chromosomes (12 + X), making it the second largest *Curculionidae* genome assembled to date, closely behind the unpublished *Trigonoscuta pilosa* (2.3 Gb) genome. By incorporating high-quality long-read gene transcripts, we initially generated 19,508 gene models. Additionally, this data set provides a high-quality reference weevil genome for use in comparative genomics and evolutionary studies for a critical group of insects that lack genomic resources.

## Methods and Results

### Insect collections

Adult pecan weevils used for genome assembly were collected alive from Circle traps established in a pecan orchard in Comanche County, Texas, USA. One female weevil collected on September 5, 2020, was used for HiFi sequencing and a second female was used for Hi-C scaffolding. Weevils used for annotation were collected from the same orchard on September 19, 2023, and included two adult males and two adult females. Weevils were sexed, dissected, and their bodies were partitioned to include head and thorax samples for both sexes to use in RNA sequencing.

### HiFi data generation

High molecular weight (HMW) DNA was extracted from the entire body of a single adult female pecan weevil (icCurCary2), collected as described above, using the Qiagen MagAttract HMW DNA Kit (Qiagen, Hilden, Germany). DNA shearing was performed using the Diagenode Megaruptor 2 (Diagenode Inc., Denville, NJ, USA) with the 20 Kb fragment protocol. The sheared DNA was prepared for PacBio HiFi sequencing using the SMRTbell Express Template Prep Kit 2.0 (Pacific Biosciences, Menlo Park, CA, USA). The final library was generated using a size-limiting SPRI-bead cleanup to remove library molecules smaller than 3 Kb in length. Sequencing was performed on a Sequel II System using Binding Kit 2.0, Sequencing Kit 2.0, and a 30-hour movie time on one SMRT cell. Raw subreads were converted to HiFi data using the Circular Consensus Sequencing (CCS) process to call a single high quality consensus sequence for each molecule with a consensus accuracy cutoff of 99.5%. The read library generated an average of 41x coverage across the genome (Fig. 4).

### Hi-C data generation

Whole body tissue from a second adult female (icCurCary2) was crosslinked using the Arima Hi-C low input protocol, and proximity ligation was performed using the Arima Genomics Hi-C Kit (Arima Genomics, San Diego, CA, USA). After proximity ligation, the DNA was sheared using a Diagenode Bioruptor (Diagenode Inc., Denville, NJ, USA) and then size-selected to enrich DNA fragments between 200–600 base pairs. An Illumina library was prepared from the sheared and size-selected DNA using the Accel-NGS 2S Plus DNA Library Kit (Swift Biosciences, Ann Arbor, MI, USA). The final Illumina Hi-C library was sequenced on an NovaSeq 6000 System (Illumina Inc., San Diego, CA, USA) at the HudsonAlpha Genome Sequencing Center (Huntsville, AL, USA).

### Genome assembly and organization

Adapter contamination was removed using HiFiAdapterFilt v1.0^17^, and the adapter-free reads (99.8% of total reads) were used as input to HiFiASM v0.14.2-r315^18^ to produce the preliminary primary pseudohaploid and corresponding alternate assemblies. Duplicated haplotigs were removed or trimmed using purge_dups v1.2.5^19^, with cutoffs automatically estimated from a generated histogram of read coverage. Purged primary sequences were added to the alternate haplotype contigs and purged of duplicates again for a final alternate assembly.

**Fig. 1:**
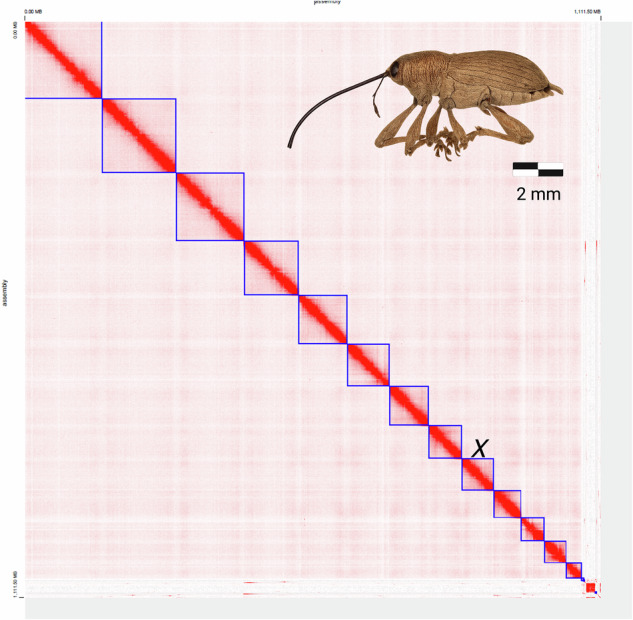
Hi-C contact map of the chromosome-level assembly of *Curulio caryae*. The x- and y-axes represent the assembled chromosomes in size order. The X chromosome is labeled. The red color indicates high contact frequencies. The strong diagonal pattern confirms accurate chromosome anchoring and assembly. A photo of *Curulio caryae* is shown in the upper right corner.

**Fig. 2:**
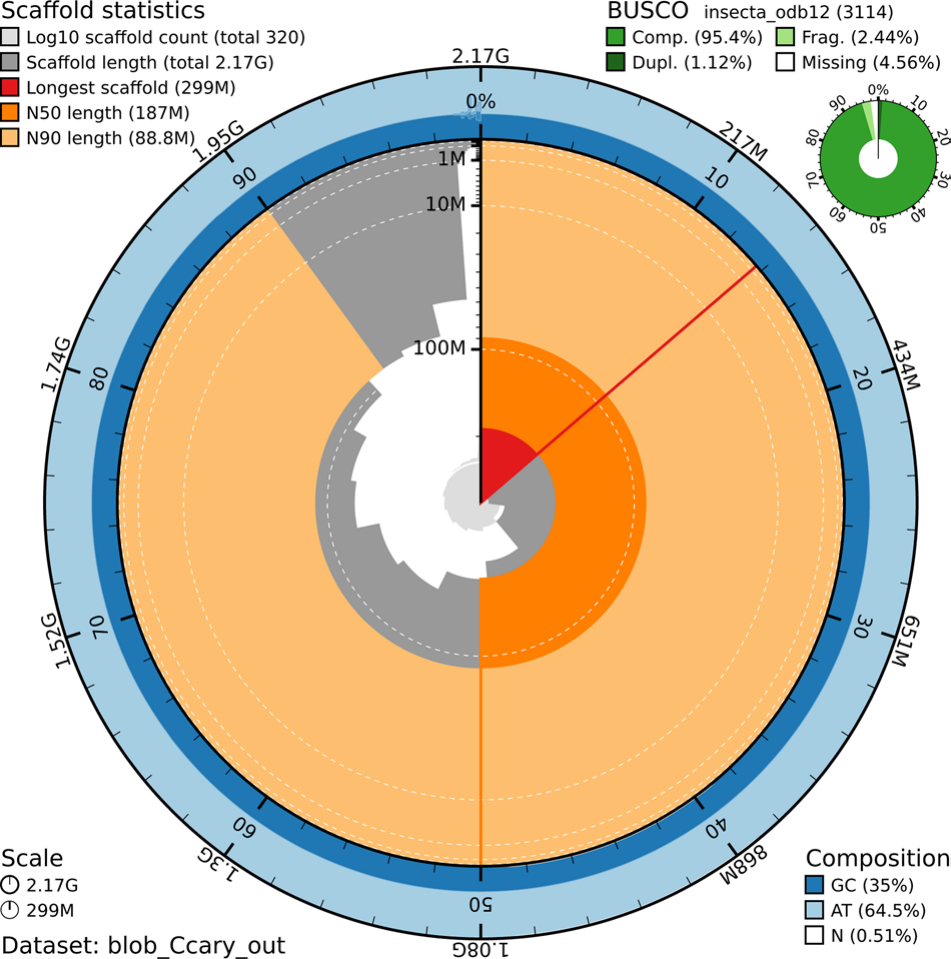
Snail plot summarizing the genome of the pecan weevil, *Curculio caryae*. The assembled scaffold count is shown in light gray in the center of the plot and the dark gray blocks refer to the length of each scaffold on the plot scale line. The longest scaffold is represented by the red line and the red region refers to its coverage percentage of the whole assembly. The N50 and N90 metrics are shown in dark and light orange blocks, respectively. GC and AT contents are summarized in shades of blue and described as Composition, bottom right corner. The Scale refers to the length of the entire assembly (2.2 G) and the longest scaffold before Hi-C (299 M). BUSCO completeness analysis is visualized in the top right corner.

YaHS v1.0^20^ (https://github.com/c-zhou/yahs) with default error correction was used to scaffold the primary assembly after aligning the Hi-C data with the ArimaHi-C Mapping Pipeline (https://github.com/ArimaGenomics/mapping_pipeline). The Hi-C contact map was then manually curated with Juicebox v1.11.08^21,22^. Autosomes were ordered according to size, and the X chromosome was identified by mapping all the pseudo-chromosomes to the X chromosome of the European acorn weevil, *Curculio glandium* (https://www.ncbi.nlm.nih.gov/datasets/genome/GCA_965648435.1/) using minimap2^23^ with default settings and ranking the scaffolds by largest percent of synteny. The ninth largest chromosome (chromosome 9) in the pecan weevil genome had the most synteny with the acorn weevil reference, >17 Mb, therefore we determined it to be the X chromosome in *C. caryae*. After manual curation of the Hi-C contact map (Fig. 1), scaffolds were checked again for contaminants using BlobTools v1.1.1^24^. Contaminants were first summarized using BlobBlurb (https://github.com/sheinasim/blobblurb) and all reads not identified as “arthropod” were removed from the read dataset with FastaParser (https://github.com/sheinasim/FastaParser). Secondly, additional contaminants were removed from the draft assembly using the BEDtools maskfasta function^25^. The final genome assembly is 2.1 gigabases in length, comprising 312 scaffolds (Fig. 2). Of these scaffolds, 112 are >50 kb in size. Moreover, the largest 13 linkage groups, plus one small contig, possess all the single copy orthologs and were further divided into 12 autosomes and one X-chromosome, as per prior karyotypic knowledge^26^ (Figs. 1, 2). This final size of the assembly is within 3% of the estimated genome size based on flow cytometry analysis (Table 1).

**Table 1:** Genome statistics for the pecan weevil, *Curculio caryae*.

Genome Metrics	
Contig/scaffold total	1214/320
Scaffold assembly sequence size	2158.808 MB 0.506% gap
N50 (contig/scaffold)	132/5 MB
L50 (contig/scaffold)	4.816/186.898 MB
Max length (contig/scaffold)	27.827/299.325 MB
Number of scaffolds >50 KB	112
% main genome in scaffolds >50 KB	99.83%
**Mean genome size estimate (flow-cytometry)**	
Male (n = 5)	2092 (±21) MB
Female (n = 5)	1963 (±13) MB

Repeat content of the pecan weevil genome was determined by generating a *de novo* pecan weevil specific library with RepeatModeler v2.0.5 (http://www.repeatmasker.org/RepeatModeler.html), followed by masking and annotation of these repeats using RepeatMasker v4.1.5 (http://www.repeatmasker.org/). Repeat content is extensive in the thirteen putative chromosomes ranging from 76%–86% ($\mu$ = 82.67% ± 3.06, Table 2).

**Table 2:** Chromosome size, predicted gene content, and percentage masked as repeats.

Chromosome	Size in nucleotides (MB)	Number of Gene Models (Braker + InterProScan)	Repeat Elements (n)	Repeat Elements (MB & percentage)
Chromosome1	299325090	1892	663516	258996719 & 86.5%
Chromosome2	286575375	1598	653815	244053907 & 85.2%
Chromosome3	261115320	1703	591632	221318115 & 84.8%
Chromosome4	210465472	1328	482844	179363811 & 85.2%
Chromosome5	186897725	1231	438462	156330620 & 83.6%
Chromosome6	161745243	1464	349476	129772248 & 80.2%
Chromosome7	153153723	1180	358144	126655350 & 82.7%
Chromosome8	127773812	982	296058	105126878 & 82.3%
Chromosome9 (X)	121760993	879	253998	104117629 & 85.5%
Chromosome10	106834911	1113	250236	83562585 & 78.2%
Chromosome11	88783051	613	220764	74499637 & 83.9%
Chromosome12	83884108	715	197694	64346169 & 76.7%
Chromosome13	57866031	700	134715	46118048 & 79.7%

### Mitochondrial genome assembly

**Fig. 3:**
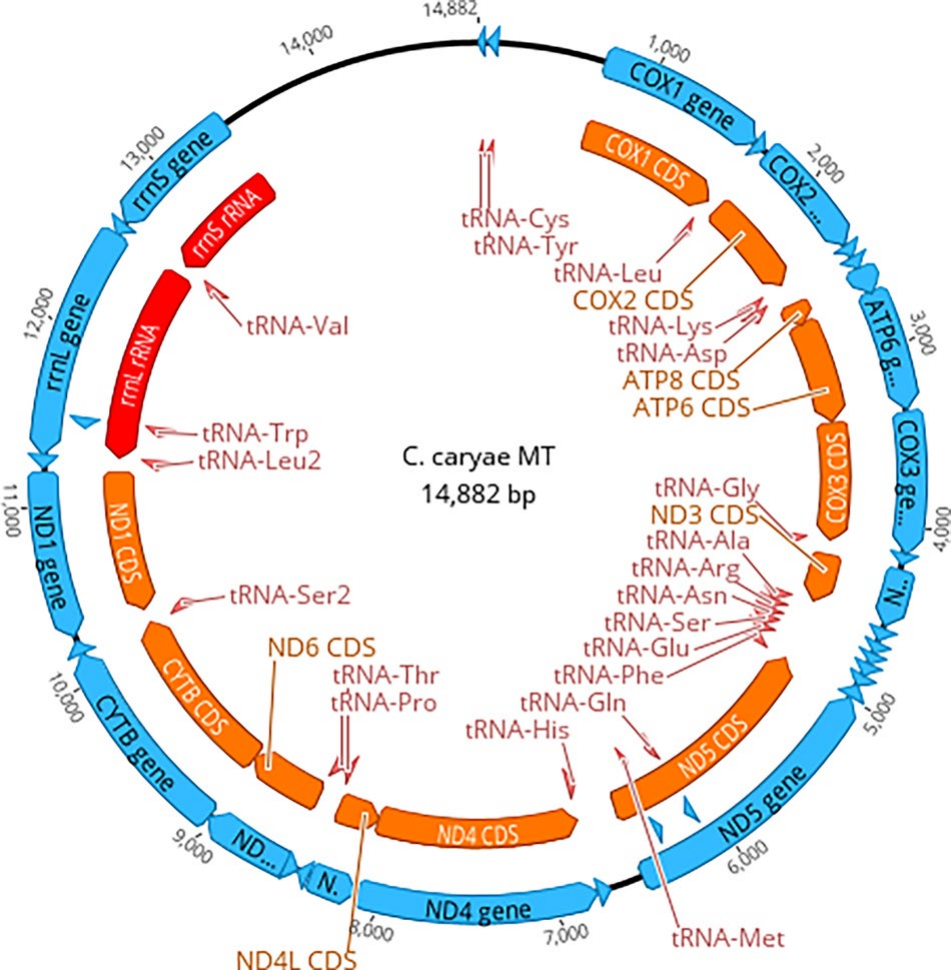
The pecan weevil, *Curculio caryae*, mitochondrial genome. Blue arrows indicate genes on the positive strand, while red arrows are for genes on the negative strand.

The mitochondrial genome was identified and annotated using MitoHiFi v2.0 (https://github.com/marcelauliano/MitoHiFi) against the *Curculio elephas* mitochondria using the long-read HiFi sequencing output (Unpublished sequence, https://www.ncbi.nlm.nih.gov/nuccore/KX087269.1). The read sequence that included the complete mitochondrial gene set was retained and circularized (Fig. 3; Table 3).

**Table 3:** Mitochondrial genes and coordinates for the pecan weevil, *Curculio caryae*.

Gene	Start	End	Strand (+/−)
tRNA-Phe	1	68	+
tRNA-Glu	69	133	−
tRNA-Ser2	134	200	−
tRNA-Asn	201	268	−
tRNA-Arg	267	330	−
tRNA-Ala	343	409	−
ND3	419	745	−
tRNA-Gly	773	835	−
COX3	835	1617	−
ATP6	1623	2297	−
ATP8	2291	2449	−
tRNA-Asp	2450	2516	−
tRNA-Lys	2517	2586	−
COX2	2588	3253	−
tRNA-Leu2	3275	3339	−
COX1	3335	4879	−
tRNA-Tyr	4872	4935	+
tRNA-Cys	4938	5002	+
tRNA-Trp	5002	5065	−
ND2	5064	6026	−
tRNA-Met	6075	6143	−
tRNA-Gln	6143	6211	+
tRNA-Ile	9093	9157	−
rrnS	11490	12271	+
tRNA-Val	12273	12338	+
rrnL	12339	13633	+
tRNA-Leu	13635	13700	+
ND1	13723	14652	+
tRNA-Ser	14670	14740	−
CYTB	14740	15876	−
ND6	15880	16374	−
tRNA-Pro	16383	16448	+
tRNA-Thr	16449	16512	−
ND4L	16521	16808	+
ND4	16936	18138	+
tRNA-His	18140	18203	+
ND5	18252	19916	+

### Genome annotation

RNA was isolated from the head and thorax segments of one adult male and one adult female pecan weevil using the NucleoMag RNA Kit (Macherey-Nagel, Düren, Germany, 744350.1) according to kit protocols. Isolated RNA was processed into PacBio Kinnex sequencing libraries using the Iso-Seq express 2.0 kit (Pacific Biosciences, Menlo Park, CA, USA 103-071-500) and Kinnex full-length RNA kit (Pacific Biosciences, Menlo Park, CA, USA,103-072-000). The prepared library was bound and sequenced at the USDA-ARS Veterinary Pest Genetics Research Unit in Kerrville, Texas, on two Pacific Biosciences SMRT cell trays with a Revio system (Pacific Biosciences, Menlo Park, CA, USA, 102-202-200) beginning with a 2-h pre-extension followed by a 30-h movie collection time. After sequencing, circular consensus sequences from the PacBio Sequel Revio subreads were obtained using the SMRTLink v13.0 software. Reads were subsequently mapped to the repeat-masked genome assembly using minimap2 with arguments for spliced nucleotide sequences (*-ax splice:hq*) to generate sam mapping files^27^. These were then compressed into bam files using samtools view -bS and used as input for gene model prediction with the Braker version 3.0.8 program^27^ (https://github.com/Gaius-Augustus/BRAKER), generating 72,879 gene models. These gene models and amino acid protein predictions were further curated for functional annotation, generating gene ontologies and protein domains using InterProScan-5.73-104.0 with PANTHER-19.0 and Pfam-37.2 databases (https://github.com/ebi-pf-team/interproscan). This yielded a final gene set of 19,508 genes with annotated functional domains.

## Data Record

The raw sequencing data, genome assembly, transcripts, and mitochondrial genome of *Curculio caryae* have been deposited at the National Center Biotechnology under BioSample SAMN26453069, accession number PRJNA813156^28,29^ (https://www.ncbi.nlm.nih.gov/bioproject/?term=PRJNA813156) and at the National Ag Library https://hdl.handle.net/10779/USDA.ADC.29329910^30^ (https://hdl.handle.net/10779/USDA.ADC.29329910). The custom annotations can be found at the National Ag Library 10.15482/USDA.ADC/30234490^31^ (10.15482/USDA.ADC/30234490).

## Technical Validation

**Fig. 4:**
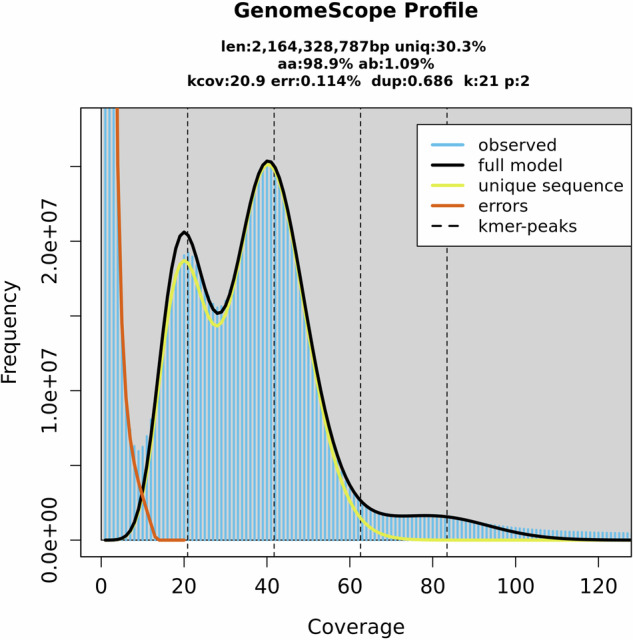
K-mer (k = 21) distribution map of *Curculio caryae* genome.

The quality of the genome was analyzed with *k-mer* analysis using GenomeScope 2.0^32^ with a coverage cutoff of 10 M. The genome size was estimated from the filtered HiFi reads using a 21-mer histogram generated by KMC^33,34^. The plot shows a main peak, near 40 with very few duplicated *K-mers* (Fig. 4). Flow cytometry was also used to confirm the genome size (Table 1) using five male and five female adult weevils. Weevils were collected from pecan tree traps, flash frozen, and kept at −80 °C until used. The brain, leg, and muscle tissues were used to estimate genome size, with all tissues producing the same estimates. The average genome size for males was 1,963 +/− 13 Mbp and 2,092 +/− 21 Mbp for females. These estimates are very similar to our final assembly size based on the 21-mer histogram at 40x coverage.

BUSCO v6.0.0 and the insecta v12 orthodb gene set was used to assess the completeness of the assembly with the metaeuk gene-predictor software^35^. The assembly is very complete with a BUSCO score of 95.4% [[S:94.3%,D:1.1%],F:2.4%,M:2.1%,n:3114], and all single copy orthologs represented on the 13 largest pseudo chromosomes in our assembly (Figs. 1, 2; Table 1).

## Data Availability

The raw sequencing data, genome assembly, transcripts, and mitochondrial genome of *Curculio caryae* have been deposited at the National Center Biotechnology under project number PRJNA813156 and at the National Ag Library https://hdl.handle.net/10779/USDA.ADC.29329910. The custom annotations can be found at the National Ag Library 10.15482/USDA.ADC/30234490.

## Code availability

Data processing was executed using published programs and default parameters unless otherwise specified in the Methods section. No custom code was used for these analyses.
